# Association between alpha 1-antitrypsin levels and intracranial aneurysms: a case-control study

**DOI:** 10.1055/s-0046-1820525

**Published:** 2026-05-12

**Authors:** Jefferson Rosi Júnior, João Paulo Mota Telles, Laís Silva Santana, Julia Leal Bassan, Juan Pedro Giraldo, Nicollas Nunes Rabelo, Eberval Gadelha Figueiredo

**Affiliations:** 1 Universidade de São Paulo, Faculdade de Medicina, Departamento de Neurologia, Serviço de Neurocirurgia, São Paulo SP, Brazil.; 2 Universidade de São Paulo, Faculdade de Medicina, Departamento de Neurologia, São Paulo SP, Brazil.; 3 Universidade de São Paulo, Faculdade de Medicina, São Paulo SP, Brazil.; 4 Barrow Neurological Institute, Division of Neurosurgery, Phoenix AZ, United States.

**Keywords:** alpha 1-Antitrypsin, Intracranial Aneurysm, Aneurysm, Ruptured, Vasospasm, Intracranial

## Abstract

**Background:**

Alpha 1-antitrypsin (A1AT) helps maintain vascular-wall integrity, but its role in the formation and rupture of intracranial aneurysms is unclear.

**Objective:**

To compare A1AT levels between patients with ruptured and unruptured intracranial aneurysms and assess the associations with vasospasm and functional outcomes.

**Methods:**

We retrospectively analyzed the medical records of patients who had serum A1AT levels measured between 2018 and 2022 during routine outpatient evaluations. The sample included 233 patients with unruptured intracranial aneurysms, 114 with ruptured aneurysms, and 55 controls with intracranial arteriovenous malformations (AVMs), but without aneurysms.

**Results:**

The mean A1AT levels were 134.9 ± 23.3 mg/dL in the unruptured group, 132.1 ± 20.3 mg/dL in the ruptured group, and 132 ± 19 mg/dL in the controls. The A1AT levels did not exhibit a significant association with the presence of intracranial aneurysms when compared to the levels of the control group (odds ratio [OR]: 1.07; 95%CI: 0.93–1.25; *p* = 0.40) or with aneurysm rupture (OR: 0.94; 95%CI: 0.84–1.04; *p* = 0.27) when compared to the levels of the unruptured group. Similarly, there were no statistically significant correlations between the protein concentrations and vasospasm (OR: 0.94; 95%CI: 0.69–1.22; *p* = 0.67) or functional outcomes defined by a score ≤ 2 on the modified Rankin Scale (OR: 0.77; 95%CI: 0.51–1.27; *p* = 0.23).

**Conclusion:**

The A1AT levels did not differ between the groups with ruptured and unruptured aneurysms, neither were they associated with vasospasm nor functional outcomes.

## INTRODUCTION

Intracranial aneurysms are significantly prevalent worldwide, and they represent a substantial clinical concern due to potential associated complications. 1 Characterized by pathological dilations in the cerebral vascular walls, these aneurysms are notable for the risk of cataclysmic cerebrovascular events if ruptured. 2 Their development is influenced by hemodynamic and structural factors, particularly at arterial bifurcations, where degeneration of the internal elastic lamina may occur. 3 4 Regions exposed to elevated wall shear stress, such as bifurcations or sharply-curved arterial segments, are especially susceptible to endothelial injury and progressive weakening of the vessel wall. 5 Moreover, anatomical variations such as hypoplastic arterial bifurcations and acute angles can further disturb flow patterns, promoting vascular inflammation and wall instability. 6 7

The ZZ genotype of alpha 1-antitrypsin (A1AT) is associated with A1AT deficiency, a protease inhibitor primarily synthesized in the liver and responsible for regulating the degradation of proteins in the bloodstream. 8 9 This genotype has been linked to various dysfunctions, including chronic obstructive pulmonary disease (COPD) in the lungs and liver fibrosis. 10 11 However, the relationship between the intracellular polymerization of A1AT and intracranial aneurysms remains inconclusive. 12 The systemic consequences of A1AT deficiency can indirectly heighten the vulnerability to aneurysms. Reduced antiprotease activity in A1AT deficiency disrupts the balance between neutrophil elastase and its inhibitor, resulting in extracellular-matrix degradation and amplified inflammatory signaling. This imbalance has been consistently associated with vascular-wall fragility in aneurysmal disease. 13 Hence, understanding A1AT dysfunction may provide valuable insights to clarify the pathogenic mechanisms involved in aneurysm formation.

The pathological insufficiency of A1AT is extensively studied, 14 15 16 with clinical manifestations frequently including emphysema, jaundice, and hepatic injury. These effects result from an imbalance in protease inhibition within the body. 17 18 19 Nevertheless, the relationship between the concentrations of this enzyme and the progression of intracranial aneurysms lacks evidence in the literature. Therefore, the aim of the present study is to compare A1AT levels in patients with ruptured and unruptured aneurysms relative to a control group.

## METHODS

The current is a retrospective case-control study involving participants from the Outpatient Cerebrovascular Neurosurgery Clinic at Hospital das Clínicas da Universidade de São Paulo (HCFMUSP). Ethical approval for the research project was granted by the local Institutional Review Board (under #61719416.6.0000.0068), and informed consent was not required due to the study's retrospective design.

The eligibility criteria encompassed patients with at least one intracranial aneurysm, whether ruptured or unruptured, who had A1AT levels recorded. The control subjects consisted of patients with cerebral arteriovenous malformation (AVM) who also had documented A1AT serum levels. This control group was chosen to ensure clinical and methodological comparability, as AVMs undergo the same diagnostic workflow, perioperative evaluation, and laboratory processing as aneurysm cases in our center. This approach minimized selection bias and reduced confounding related to laboratory assessment. Consequently, other cerebrovascular conditions, including non-aneurysmal subarachnoid hemorrhage, dural arteriovenous fistulae, and vascular lesions, which are managed outside the Neurointerventional Service, were excluded because they present distinct pathophysiological mechanisms and more heterogeneous laboratory profiles. Patients presenting simultaneously with aneurysms and AVMs were excluded. Subjects with conditions known to alter serum A1AT levels, including chronic liver disease, COPD, and chronic inflammatory disorders, were also excluded to minimize confounding.

Only variables documented systematically and uniformly across the aneurysm and control groups were incorporated into the dataset, and parameters not consistently available in the medical records were not included. The study's data variables included patient age, sex, aneurysm location, comorbidities, previous rupture events, vasospasm occurrence, and assessment of functional outcomes using the modified Rankin scale (mRS). Aneurysm rupture was defined by the presence of subarachnoid hemorrhage on a computed tomography scan performed upon admission, accompanied by clinical presentation compatible with acute bleeding. Vasospasm was determined through digital subtraction angiography performed during the acute phase of the subarachnoid hemorrhage, defined in the present study as the period up to 21 days after hemorrhage, when clinically indicated, and it was classified according to the severity described in the angiographic report.

Potential confounders, including age, sex, hypertension, smoking status and other cardiovascular risk factors, were retrieved from the medical records and compared among groups. These variables were incorporated into the descriptive analysis to assess baseline comparability. The laboratory examinations initially focused on measuring A1AT levels in patients with and without aneurysms, both ruptured and unruptured, including a control group. At our center, A1AT measurement is routinely included in the preoperative laboratory panel rather than requested on clinical grounds, which minimizes indication bias and preserves group comparability. All serum samples from patients with ruptured aneurysms were collected at least 1 month after discharge to ensure the stability of the A1AT measurements and minimize the effects of the acute inflammatory state. Regular serum assays were performed, and the serum A1AT concentrations were measured in the institution's central laboratory using immunonephelometry, under standardized processing conditions and manufacturer-defined reference ranges.

### Statistical analysis

Categorical data are represented by frequency and the corresponding valid percentages. The normality of the continuous data was assessed through the Shapiro-Wilk's test. Data following a normal distribution were expressed as mean and standard deviation values, whereas non-normally distributed data were expressed as median and interquartile range (IQR) values.

Continuous data analysis employed the *t* -test for normal distributions, and the Mann-Whitney U test for non-normal ones, while categorical data comparisons used the Chi-square test. The associations between A1AT levels and ruptured aneurysms, as well as between vasospasm and functional outcome, were assessed using uni- and multivariable logistic regression analyses. Statistical significance was defined as *p* -value < 5%, with tests performed employing a bicaudal approach. All analyses were performed using the R (R Foundation for Statistical Computing, 4.0, Vienna, Austria) software.

## RESULTS

### Patient characteristics

A total of 402 patients were included: 233 with unruptured aneurysms, 114 with ruptured aneurysms, and 55 control subjects. Patients with unruptured aneurysms had a slightly higher mean age (56.6 years) compared with the ruptured group (53.9 years). There was a predominance of female subjects: 70.9% of the controls, 73% of the unruptured group, and 76.3% of the ruptured group.

Comorbidities such as smoking and dyslipidemia were more frequent in the control group, whereas hypertension showed similar distribution across groups. Most patients in the aneurysm groups presented with three or fewer aneurysms. The baseline characteristics were otherwise similar across groups, with significant differences only in age and dyslipidemia ( *p* = 0.02), as shown in Table 1 .

**Table 1:** Baseline characteristics of the study groups

	Unruptured aneurysm (n = 233)	Ruptured aneurysm (n = 114)	Controls (n = 55)	*p* -value
Mean age (years)	56.6 ± 13.1	53.9 ± 13.9	52.6 ± 13.9	**0.02**
Female sex: n (%)	170 (73)	87 (76.3)	39 (70.9)	0.71
Smoker: n (%)	63 (27)	27 (23.7)	16 (29.1)	0.71
Hypertension: n (%)	64 (27.5)	30 (26.3)	19 (34.5)	0.51
Previous stroke: n (%)	8 (3.4)	3 (2.6)	1 (1.8)	0.79
Dyslipidemia: n (%)	48 (20.6)	27 (23.7)	21 (38.2)	**0.02**
Number of aneurysms: median (interquartile range)	1 (1)	1 (1)	0	0.65
Mean level of alpha 1-antitrypsin (mg/dL)	134.9 ± 23.3	132.1 ± 20.3	132 ± 19	0.45

Notes: The values of *p* refer to analysis of variance (ANOVA) or Chi-squared tests, as appropriate; *p* -values in bold indicate statistical significance.

### Alpha 1-antitrypsin

Table 2 displays the odds ratios (ORs) regarding the associations involving serum A1AT levels and the presence of aneurysms, rupture status, vasospasmm and functional outcomes, in uni- and multivariable analyses.

**Table 2:** Association between alpha 1-antitrypsin levels and the presence of aneurysms, aneurysm rupture, vasospasm, and functional outcomes

		Odds ratio	95%CI		*p* -value
Aneurysm		1.05	0.92	1.21	0.53
Aneurysm rupture		0.94	0.84	1.05	0.28
Vasospasm*		0.99	0.96	1.02	0.68
Score on the modified Rankin scale ≤ 2*		0.99	0.97	1.01	0.19
*Multivariable*	Aneurysm	1.07	0.93	1.25	0.40
	Aneurysm rupture	0.94	0.84	1.04	0.27
	Vasospasm*	0.94	0.69	1.22	0.67
	Score on the modified Rankin scale ≤ 2*	0.77	0.51	1.27	0.23

Notes:S The multivariable logistic regression models were adjusted for age and dyslipidemia. *Only ruptured aneurysms.

**Figure 1:**
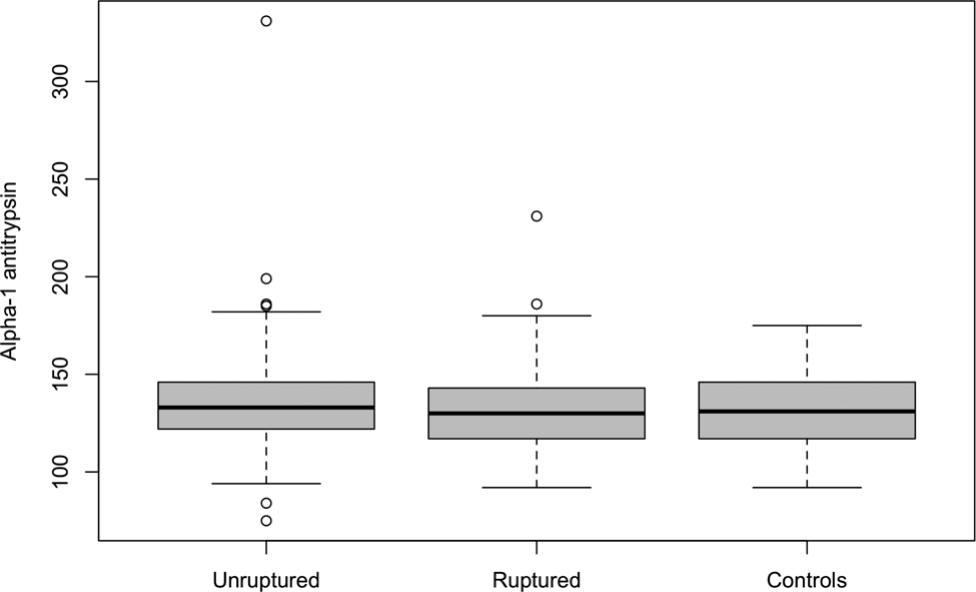
Distribution of alpha 1-antitrypsin among the study groups.

The unruptured group presented a mean A1AT level of 134.9 ± 23.3 mg/dL, while the ruptured group presented a mean level of 132.1 ± 20.3 mg/dL. The control group's mean A1AT level, of 132 ± 19 mg/dL, closely matched that of the ruptured group. Figure 1 features a boxplot illustrating a comparison of the AIAT levels of the aneurysm groups and the controls.

The statistical assessment revealed no significant association regarding A1AT levels and aneurysm occurrence or rupture. The uni- (OR: 1.05; 95%CI: 0.92–1.21; *p* = 0.53) and multivariable analyses (OR: 1.07; 95%CI 0.93–1.25; *p* = 0.40) evidenced no statistically significant association between A1AT levels in aneurysm patients compared to the controls.

The univariate (OR: 0.94; 95%CI: 0.84–1.05; *p* = 0.28) and the age and dyslipidemia-adjusted multivariable (OR: 0.94; 95%CI: 0.84–1.04; *p* = 0.27) analyses found no significant association between higher A1AT levels and aneurysm status (ruptured or unruptured). No significant correlation was observed between higher A1AT levels and vasospasm in ruptured aneurysms, as per the uni- (OR: 0.99; 95%CI: 0.96–1.02; *p* = 0.68) and multivariable (OR: 0.94; 95%CI: 0.69–1.22; *p* = 0.67) models. Similarly, the uni- (OR: 0.99; 95%CI: 0.97–1.01; *p* = 0.19) and multivariable analyses (OR: 0.77; 95%CI: 0.51–1.27; *p* = 0.23) lacked a significant association between A1AT levels and functional outcomes (mRS ≤ 2).

## DISCUSSION

The findings of the current study showed no significant differences in circulating A1AT levels between patients with and without intracranial aneurysms, nor between ruptured and unruptured cases. Neither did the A1AT concentrations correlate with angiographic vasospasm nor functional outcomes, indicating limited discriminatory or prognostic value for this biomarker in this cohort.

These results contrast with those of earlier phenotype-based observations 20 21 22 23 24 suggesting a contribution of impaired antiprotease activity to aneurysmal remodeling, including reports of altered elastase-A1AT balance and higher frequencies of deficient *serpin family A member 1* ( *SERPINA1* ) alleles among patients with aneurysmal disease. Additional evidence has suggested that the atypical and severely-deficient genotypes may be overrepresented in aneurysm populations. Although these studies provide mechanistic plausibility for a link between A1AT dysfunction and vascular fragility, they did not consistently demonstrate differences in circulating A1AT levels, which aligns with the absence of serum-level variation in our data. Evidence of protease–antiprotease imbalance has also been documented, with studies 20 21 demonstrating disproportionate elastase activity relative to A1AT in patients with intracranial aneurysms, supporting a mechanism of extracellular-matrix degradation and vascular-wall weakening.

One explanation for the lack of detectable differences lies in the disconnect between serum A1AT concentrations and genotype- or tissue-level dysfunction. Variants such as the Z allele may undergo intracellular polymerization, impair secretion, and promote local protease dysregulation, even when systemic levels fall within reference ranges. Local vascular susceptibility, inflammatory milieu, and genetic background may, therefore, exert stronger influence than circulating A1AT. The fact that A1AT was measured at least 1 month after rupture, a time chosen to avoid acute-phase influences, may also attenuate true biological contrasts.

A brief pathobiological framework helps contextualize these findings. Aneurysm formation and progression reflect the interaction of hemodynamic stress, extracellular-matrix remodeling, inflammation, and oxidative injury, processes in which protease–antiprotease imbalance plays a contributory rather than an isolated role. Under these conditions, systemic A1AT levels may be insufficient to capture the complexity of local vascular events. Lifestyle and environmental exposures, particularly tobacco consumption, 10 25 26 27 can further alter the functional expression of A1AT deficiency, reinforcing the multifactorial nature of aneurysmal biology.

### Limitations

The limitations of the current study include variations in serum A1AT levels among patients, depending on the inflammatory state, the studied population, and the genetic profile. 28 Therefore, the generalization of our results to other populations may be restricted. Another limitation relates to the time of A1AT collection in patients with ruptured aneurysms: samples were obtained at least 1 month after discharge to avoid acute-phase inflammation, but this delay may attenuate true biological differences between ruptured and unruptured aneurysms, potentially reducing detectable contrasts. A further constraint is the absence of detailed aneurysm descriptors, such as size, morphology, family history, and validated risk scores, which were not consistently-available in this retrospective dataset and therefore did not enable correlations between A1AT levels and anatomical or risk-based parameters. Severity grading with the Fisher or modified Fisher scales was likewise unavailable for several cases of ruptured aneurysm, precluding a stratified analysis of rupture severity and its relationship with A1AT levels. Despite such limitations, the present represents the first extensive sample study to compare A1AT levels among patients with ruptured and unruptured aneurysms and a control group.

In conclusion, A1AT levels were not elevated in the unruptured group compared to the ruptured group. Furthermore, there were no discernible differences in A1AT levels between the control group and the aneurysm groups. Lastly, A1AT levels did not exhibit any correlation with vasospasm nor functional outcomes in this cohort. Despite the negative results, the study's findings clarify the limited role of biomarkers such as A1AT in intracranial-aneurysm outcomes. Future research may focus on specific populations or investigate the role of other biological factors in the relationship between A1AT levels and intracranial aneurysms.
